# Da vinci robotic-assisted treatment of pediatric choledochal cyst

**DOI:** 10.3389/fped.2022.1044309

**Published:** 2022-11-01

**Authors:** Shan Chen, Yang Lin, Di Xu, Jianli Lin, Yunlong Zeng, Lizhi Li

**Affiliations:** ^1^ Clinical Laboratory Department, Fuzhou Second Hospital Affiliated to Xiamen University, Fuzhou, China; ^2^ Department of Pediatric Surgery, Shengli Clinical Medical College, Fujian Medical University, Fuzhou, China; ^3^ Laboratory Provincial Clinical Medical College, Fujian Medical University, Fuzhou, China

**Keywords:** choledochal cyst, da vinci robot, laparoscopy, pediatrics, postoperative care

## Abstract

**Objective:**

To evaluate the advantages and disadvantages of da Vinci robot and laparoscopy in treating pediatric choledochal cysts.

**Methods:**

We retrospectively analyzed clinical data from forty-two children diagnosed with choledochal cysts in our hospital from January 2018 to January 2021. Twenty children underwent da Vinci robotic surgery, and the others underwent traditional laparoscopy. We compared differences in general information and preoperative, intraoperative, and postoperative differences between the two surgical groups.

**Results:**

There was no statistically significant difference in age, gender, weight, type, maximum cyst diameter, preoperative C-reactive protein (CRP) value, postoperative complication rate, and postoperative pain score between the two surgical groups (*P *> 0.05). The average age of the robot-assisted group was 3.62 ± 0.71 years old (range = 1–12 years). There were nineteen cases of Todani type I, one patients of other types, and the maximum cyst diameter was 35.45 ± 9.32 mm (range = 12–56 mm). In the laparoscopic group, the average age was 3.08 ± 0.82 years old (range = 3–10 years). Twenty-one patients had Todani type I, and one had other types. The maximum cyst diameter was 31.94 ± 8.64 mm (range = 10–82 mm). The robot-assisted group had better abdominal drainage, postoperative CRP value, fasting time, and discharge time than the laparoscopic group (*P *< 0.05).

**Conclusion:**

Compared with laparoscopy, the da Vinci system has the advantages of less tissue damage, faster recovery, and better healing in the treatment of children with congenital choledochal cysts. With technological advancements and an increased number of experienced surgeons, robotic surgery may become a new trend in surgery.

## Introduction

Choledochal cyst is a rarely congenital biliary malformation. It predominantly affects the Asian population with an incidence of 1 in 1000 live births, compared to an incidence of 1 in 100000–150000 live births in the western population (1). The most common classification method for choledochal cysts is Todani typing, which is divided into 5 types, of which type I accounts for about 80% of all cases, exhibiting the cystic or fusiform dilation of the extrahepatic bile ducts (2). Although choledochal cyst is a benign lesion, it has the potential to become malignant and may develop into other related diseases (such as cholangiocarcinoma, choledocholithiasis, cholangitis and pancreatitis, etc.) (3). Due to the above mentioned risks involved, prompt treatment is essential.

The main treatment methods are cholecystectomy, choledochocystectomy, and standard Roux-en-Y hepaticojejunostomy. In recent years, the operation style has developed from laparotomy, laparoscopy, to robotic-assisted surgery.The first use of laparoscopy to treat congenital choledochal cysts is implemented by Farello et al. in 1995 (4). In 2006, Woo et al. has reported the first and successful case of a 5-year-old child who underwent a type I choledochal cyst resection under the assistance of robotic laparoscopy in the world (5). Until now, more than 20 general hospitals and children's hospitals in China have successfully performed the operation independently. Robotic surgery has become an important part of minimally invasive surgery in children (6).

This study aims to compare the advantages and disadvantages of laparoscopy using the da Vinci robot vs. traditional laparoscopy in the pediatric treatment of congenital choledochal cysts.

## Materials and methods

### Clinical information

We retrospectively analyzed clinical data from forty-two children diagnosed with choledochal cysts in our hospital from January 2018 to January 2021. Twenty cases underwent da Vinci robotic surgery and were assigned as the da Vinci robotic surgical system (RSS). The other twenty-two patients underwent traditional laparoscopic treatment as the conventional laparoscopic system (CLS). The two groups of children's general condition before surgery were determined to be sufficient to proceed with laparoscopic and robotic surgery. Whether the surgical approach using da Vinci system or laparoscopy was decided between the surgeon and patient's parents according to their preferences. And the age of RSS patients was not less than one year old. Irrespective of the surgical approach used, all the operations were completed by the same treatment group with sufficient experience.

Inclusion criteria were as follows: (1) The diagnosis of the choledochal cyst was confirmed based on medical records and imaging examination results; (2) CO_2_ pneumoperitoneum could be tolerated during either surgery; (3) The child's coagulation ability was normal, and no severe organ dysfunction was evident, and (4) condition of focus was not combined with comorbidities or malformations of the digestive system. Exclusion criteria: (1) Intrahepatic bile duct stenosis, (2) intrapancreatic calculi, (3) secondary operations and (4) repeated chronic inflammation around the bile duct, (5) severe intracystic infection, (6) cyst perforation, and (7) malignant transformation of choledochal cyst before the operation.

### The location of trocars

**Figure 1:**
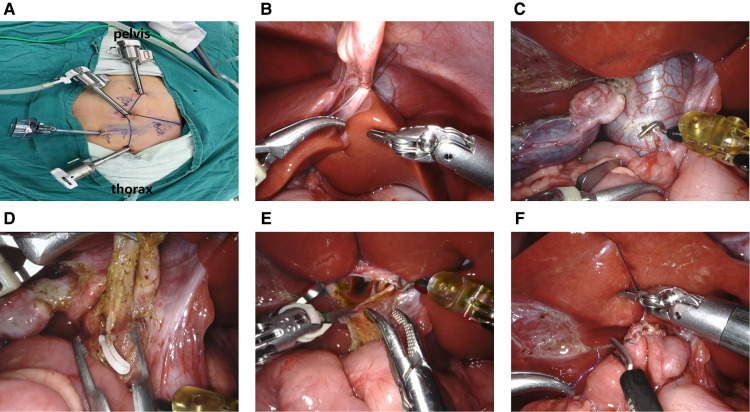
(**A**) location of robotic trocars; (**B**). Suspension of the ligament teres; (**C**). Cutting the cyst's anterior wall; (**D**). Clipping of the narrowest part of the distal end of the cyst; (**E**). Detection of the common hepatic duct with a “trumpet” shape; (**F**). Common hepatic duct jejunal anastomosis.

The general location of trocars is comprised of a 8 mm trocar around umbilicus for lens arm and two 8 mm trocars for manipulation arms, the left in midclavicular line flat costal margin while the right in midclavicular line flat lower quadrant, and the robotic surgery additionally require a 5 mm trocar for assistant operation (Figure 1A). And the distance could be lengthened as the limitation of abdominal cavity or the enlarger lesions.

### Laparoscopic choledochal cyst excision procedure

The patients were placed in the supine position, and general anesthesia was induced through an endotracheal tube. The traditional four-port laparoscopic choledochal cyst resection and Roux-en-Y hepaticojejunostomy were performed according to the method described in the following (4): laparoscopic excision of the gallbladder and common choledochal cyst, followed by the anastomosis of folded proximal jejunum and distal jejunum outside the abdominal cavity, and finally completion of endoscopic hepaticojejunostomy.

### Da vinci robotic surgery procedure

***Pre-docking stage***: We explored the abdominal cavity using standard laparoscopy. In the Treitz ligament and about 15 cm away from its distal end, the intestine was raised. After end-to-side anastomosis of the jejunum outside the abdominal cavity, the intestine was returned to the abdominal cavity.

***Docking stage***: The camera port III and operation port II, IV of Xi robotic system completed docking from the rostral (head) side of the child and the ligament teres was suspended to expose the hilar area (Figure 1B). The gallbladder and cyst were freed, the anterior wall of the cyst was incised *via* the unipolar electrocoagulation (Figure 1C), bile and stone residue were aspirated and removed, and the posterior cyst wall was gradually transected. The distal end of the cyst was cut at its thinnest diameter and ligated to the pancreatic duct (Figure 1D). The proximal end of the cyst wall was freed from the junction with the normal common hepatic duct. The confluence of the left and right hepatic ducts was identified (Figure 1E) and the cyst wall was removed. According to the common hepatic diameter, the opposite wall of the mesentery was incised, and 5–0 absorbable sutures were applied to close the hepatic duct and the inner corner of the intestinal incision. A continuous suture of the hepatic duct and the posterior wall of the jejunal incision was applied. Then, another suture was used to complete the front wall anastomosis from the inside, and the front suture was tied at the outer corner of the anastomosis (Figure 1F). The drainage tube was placed in the hepatorenal crypt. The area was rinsed to confirm that there was no intestinal torsion, bleeding, or bile leakage. The pneumoperitoneum was then decompressed and the incision was sutured closed.

### Intraoperative and postoperative observation and recording indicators

The operation time was monitored and recorded, as well as the postoperative complications, the amount of abdominal drainage fluid on the first and third days after surgery, CRP value the day after surgery, pain score, postoperative fasting time, and postoperative discharge time. Postoperative pain was measured using the modified facial expression scoring method (FLACC scoring method: including facial expression, leg movement, activity, crying, and consolability; Each behavior is worth 0 to 2 points, with a full score is 10 points, 0 points for no pain, 7 to 10 points to severe pain) (7), designed for infants and young children and Verbal Numerical Rating Scale (VNRS): 0 = no pain, 10 = most severe pain. Postoperative evaluation was performed every 6 h for 36 h post-surgery. Postoperatively, the patients were fasted until the appearance of bowel activity, and this was usually found to be the third day after surgery in both groups. Water was given first, followed by a liquid and a then soft diet. After all diets were able to be consumed by the patient without any discomfort, abdominal pain, or other complications, only then was discharge considered.

### Postoperative follow-up

The children were followed up at 1, 3, 6, and 12 months after surgery, and then every 6 months for physical examination, abdominal ultrasound, and laboratory examinations. According to clinical manifestations, abdominal ultrasound and blood biochemical examination results, postoperative biliary leakage, pancreatic leakage, anastomotic stenosis, cholangitis, pancreatitis, intestinal obstruction, wound infection and incisional hernia were evaluated. One month after the operation, upper gastrointestinal radiography was performed to determine whether the Roux biliary branch refluxed to the hepatic hilar and evaluate the degree of reflux.

### Statistical analysis

Statistical analysis was performed using SPSS 22.0 software. When measurement data conformed to a normal distribution, data were expressed as mean ± SD, and an independent sample t-test was used to compare data from the two groups. Enumeration data was expressed as rate (%), the comparison between the two groups uses the X^2^ test, and the exact X^2^ used the Fisher method. *P *< 0.05 was considered statistically significant.

## Results

Both the RSS group and the CLS group were completed as planned, and no patient was converted to laparotomy, all the peroperative bleeding were under 10 ml. No statistically significant difference in general data and preoperative conditions between the two groups of children was observed (*P* > 0.05) (Table 1). The amount of abdominal drainage on the first and third days, the postoperative CRP value, the postoperative fasting time, and the postoperative discharge time of the robot surgical group were significantly less than in the laparoscopic group (*P *< 0.05) (Table 2). There was no obvious reflux into the hepatic duct in the ascending loop of Roux-en-Y in the upper gastrointestinal radiography. All children were asked to return to the hospital for a review of abdominal color Doppler ultrasound and liver function in one, three, and six months following discharge. The median follow-up time of the robot and the laparoscopic groups were 11 months and 13 months, respectively. One patient in the robot group developed peritoneal effusion on the third day post-surgery, and the effusion was resolved after ultrasound-guided puncture and drainage. In the laparoscopic group, one child exhibited abdominal effusion after surgery, which also recovered after puncture and drainage. To date, no long-term complications has been documented in any of the children, and there was no statistically significant difference in the incidence of complications between the two groups (*P *= 0.945).

**Table 1:** Comparison of general information and preoperative conditions of the two surgical groups.

Groups			Rss	Cls	*t/X^2^*	*P* value
Sample number			20	22	–	
Sex (%)	Male (%)		6 (30.00)	7 (31.82)	0.016	0.899
	Female (%)		14 (70.00)	15 (68.18)		
Age (year, $\bar{x}$±s)			3. 62 ± 0. 71	3. 08 ± 0. 82	2.318	0.026
Weight (kg, $\bar{x}$±s)			17.63 ± 4. 21	15.91 ± 3.97	1.363	0.181
Todani type (%)		Todani I	19 (95.00)	21 (95.45)	0.005	0.945
		Other types (II and IVa)	1 (5.00)	1 (4.55)		
The preoperative CRP value (mg/L, $\bar{x}$±s)			3.13 ± 1.06	2.97 ± 0.91	0.526	0.602
The maximum cyst diameter (mm, $\bar{x}$±s)			35.45 ± 9.32	31.94 ± 8.64	1.614	0.114

Rss, Da Vinci robotic surgical system; Cls, conventional laparoscopic system; CRP, C-reactive protein; *t/X^2^*, a *χ*^2^ test with continuity correction.

**Table 2:** Comparison of intraoperative and postoperative conditions of the two surgical groups.

Groups		Rss	Csl	*t/X^2^*	*P* value
Sample number		20	22	–	
Operation time (min, $\bar{x}$±s)		285.04 ± 40.22	190.17 ± 25.05	9.271	0.000
Postoperative complications (%)	Yes	1 (5.00)	1 (4.55)	0.005	0.945
	No	19 (95.00)	21 (95.45)		
Postoperative abdominal drainage fluid volume (ml, $\bar{x}$±s)	1st day	35.00 ± 8.00	42.00 ± 9. 00	−2.653	0.011
	3rd day	21.00 ± 6.00	32.00 ± 5.00	−6.747	0.000
CRP value of postoperative day 1 (mg/l, $\bar{x}$±s)		16. 25 ± 2.84	24. 03 ± 3.49	−7.875	0.000
Pain score ($\bar{x}$±s)		0.95 ± 0.37	1.15 ± 0.41	1.653	0.016
Fasting time (d, $\bar{x}$±s)		3. 80 ± 0.70	4. 45 ± 1.25	−2.050	0.047
Postoperative discharge time (d, $\bar{x}$±s)		7.30 ± l. 10	9.70 ± 1. 80	5.140	0.000

Rss, Da Vinci robotic surgical system; Cls, conventional laparoscopic system; CRP, C-reactive protein; *t/X^2^*, a *χ*^2^ test with continuity correction.

## Discussion

The Todani I of choledochal cyst is occupied with the majority of congenital biliary dilatation (2), with the surgical recommendation of cysts resection and Roux-en-Y hepaticojejunostomy. Our study intends to summarize the efficiency of robotic surgery on Todani I and II, recovering from cysts resection, and yet, Todani IVa. However, the Todani IVa might be accompanied with intrahepatic choledochal lesions to be difficultly resolved. The sample in our study isn't intended to suffer from any specific complications after surgery. The study adopts to complete the extraperitoneal jejunal anastomosis, and then robotic anastomosis of the cyst resection and biliary intestine, which shortening the operation duration (8). The choledochal terminal is free until pancreas, and the proximal cyst is completed removed to prevent tumorigenesis (9), and the choledochal hilar stenosis is recommended to be reconstructed. The subsequently robotic Roux-en-Y anastomosis is similar to that of the laparotomy and laparoscopy, with precise anti-reflux and few long-term complications.

Robotic minimally invasive surgery is an important trend in the development of modern surgical technology, also in the treatment of pediatric choledochal cysts (10). We have begun using da Vinci robots in biliary reconstruction surgery in January 2018 and have successfully treated children with choledochal cysts. This study indicates that the robot group displayed lower postoperative CRP, reduced postoperative abdominal drainage, shorter postoperative fasting time, and shorter postoperative discharge time compared with the laparoscopic group. Robotic surgery is characterized by higher accuracy, finer anatomical operations, more comprehensive vision, and less damage to blood vessels and other critical tissues and organs. Reduced tissue damage induces less postoperative local inflammation, less exudate, and faster tissue repair and recovery of gastrointestinal function, which account for the decreased postoperative outcomes. Although the postoperative pain score in the robot group was lower than that of the laparoscopic group, the difference is not statistically significant. This may be because the FLACC scoring method and the VNRS rating scale used are subjective and may not be accurate enough in comparing these procedures. Robotic operation time is also longer, requiring more anesthesia, which could impact the postoperative analgesic effect.

Laparoscopy can only provide a two-dimensional plane of vision, which reduces three-dimensional perception during surgery. Hand-eye coordination becomes diminished, which can only be overcame by repeated practice and accumulation of clinical experience (11). In contrast, the da Vinci robotic surgery system provides a three-dimensional field of view with a magnification of up to 10 times, which can present clear, accurate and high-resolution images. An advantage of choosing da Vinci robotic surgery is the ability to perform completed resection of a choledochal cyst, which can be finely stripped down to the pancreatic segment, and the greatest extent of cyst tissue (12). The surgeon can also adjust the depth and angle of the lens according to their own preferences and requirements. In addition, the magnified view of the lower part of the common bile duct benefits for fine manipulation (13). Dissecting the hepatoduodenal ligament under a clear field of vision simultaneously can effectively avoid damage to important tissues such as the right hepatic artery and portal vein (14). One of the patients in the present study has a history of repeated infections and severe adhesions around the cyst are found during the operation. It's more efficient for da Vinci robot to separate adhesions than laparotomy or laparoscopy, accounting for the smaller manipulator, with the significant signature of flexibility, stability and precision (15). When the cyst is removed, the imaging signature clarifies the tissue structure around the cyst wall, making the separation process more precise, and effectively avoided secondary injury (16).

Laparoscopy and associated instruments provide limited freedom of movement. Meanwhile, the simulation manipulator of the da Vinci system is highly flexible, simulating the parallel movement, bending, opening, closing, and rotation of human hands (17). Rotation capabilities extend up to 540^o^ to accurately grasp, free, cut, and suture (18). The quality of biliary-enteric anastomosis during robot-assisted choledochal cystectomy is directly related to postoperative bile leakage incidence. Therefore, the reliability of each stitch is paramount. The use of robotic suturing can maximize the surgeon's hand movements for suture operation (19). For the hepatic duct and jejunum anastomosis, the artificial wrist manipulator with microtremor and filtering function affords better dexterity and a larger range of motion than traditional laparoscopic instruments, making the anastomosis process easier and more delicate. This study has showed that the incidence of patient bleeding was low and no bile leakage was observed after the operation. These positive outcomes are attributed to the preciseness of manipulator on suture, which also promotes early patient recovery under the Nursing Process Enhanced Recovery After Surgery (ERAS) concept (20). Studies have reported an average of 7.5 days of postoperative discharge for children who underwent robotic radical choledochal cystectomy (21, 22). The average postoperative hospital stay in our study is ideal at an average of 7.30 ± l.10 day.

Additionally, the laparoscope is designed to be an counter-intuitive reverse operation. When the operation time is prolonged, the surgeon is prone to fatigue in the hands, neck, shoulders, and waist, which will cause instrument to shake and reduce the accuracy of operation. In contrast, robotic surgery allows the surgeon to sit comfortably on the operating station outside the aseptic area to complete the surgery. This approach is especially suitable for long-time and complicated operations involving hepatobiliary tumors. The method by which the operation is performed is essentially the same as that of classic endoscopy, and the surgeon can master it more quickly (23). With the tacit cooperation of assistants and nurses, and the proficiency of the robot and patient docking process, operation time will be further shortened. In the present study, the average operation time of the first two children in this group is about 7 h. With improved proficiency, operation time is quickly shortened to about 5 h.

Koga et al. first reports the comparison between the laparoscopy and robot-assist surgery (24), and the da Vinci robot also has certain limitations (22). Firstly, the docking process of robot system is difficult and time-consuming. To avoid repeated docking during the operation, we first perform extracorporeal jejunum-jejunum end-to-side anastomosis, and then perform endoscopic cystectomy and anastomosis. Secondly, the cost is higher than that of laparoscopy, which limits wider clinical application. Lastly, a number of surgical limitations including longer surgical time, lack of force or haptic feedback, learning curve and training needs, limitations with instruments, operating room space, and the potential risk of clashing of surgical arms. Thus, there is a significant demand for upgrading experience of surgical teams. A long term follow-up is deemed necessary to validate the advantages of robotic surgery to suffering from these limitations. Our study is a retrospective study in a single center and so, in order to validate our findings and to investigate further the true detailed benefits of robotic-assisted surgery for the treatment of choledochal cysts, a prospective larger, multi-center clinical trial is necessary.

## Conclusion

Compared with laparoscopy, the da Vinci system has the advantages of less tissue damage, faster recovery, and better healing in the treatment of children with congenital choledochal cysts. With technological advancements and an increased number of experienced surgeons, robotic surgery may become a new trend in surgery.

## Data Availability

The original contributions presented in the study are included in the article/Supplementary Material, further inquiries can be directed to the corresponding author/s.
